# Supplementary figures and images for: Telehealth at scale can improve chronic disease management in the community during a pandemic: An experience at the time of COVID-19
Source: PLoS One. 2021 Sep 29;16(9):e0258015. doi: 10.1371/journal.pone.0258015 (PMC8480747; doi:10.1371/journal.pone.0258015)

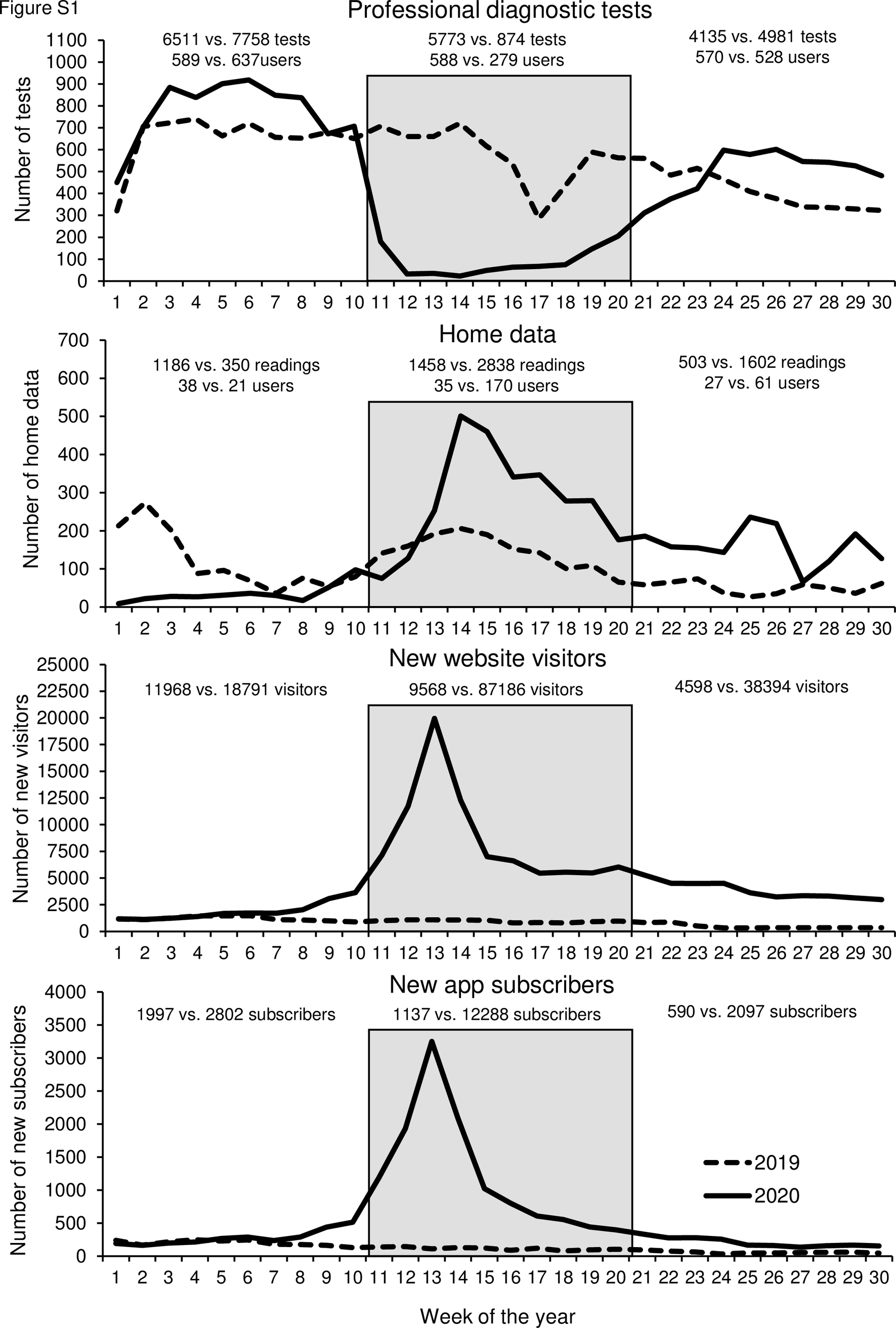

Supplement: S1 Fig — Pre-lockdown, lockdown and post-lockdown periods are identified as in Fig 1. (TIF) [file pone.0258015.s001.tif]

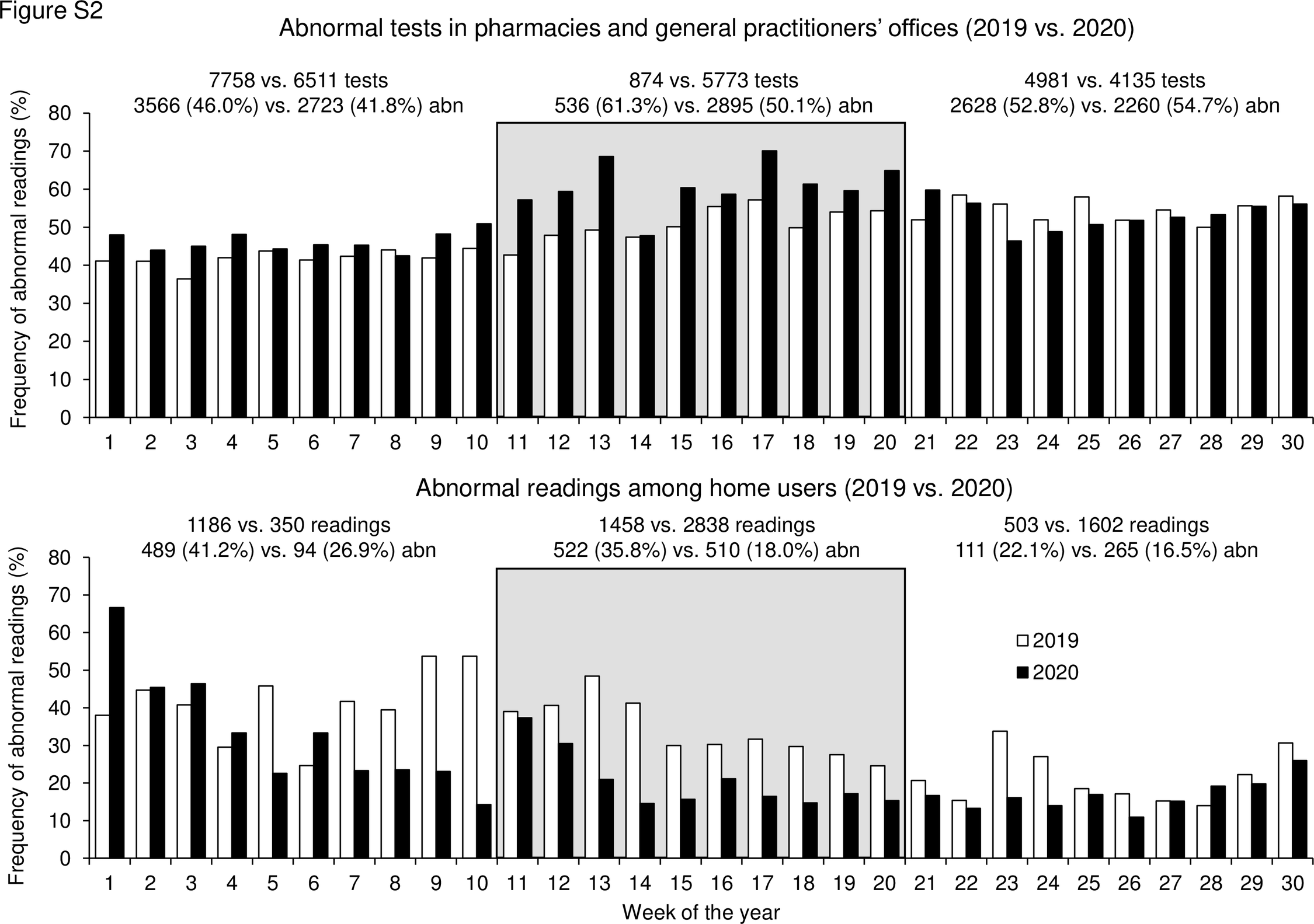

Supplement: S2 Fig — Pre-lockdown, lockdown and post-lockdown periods are identified as in Fig 2. (TIF) [file pone.0258015.s002.tif]

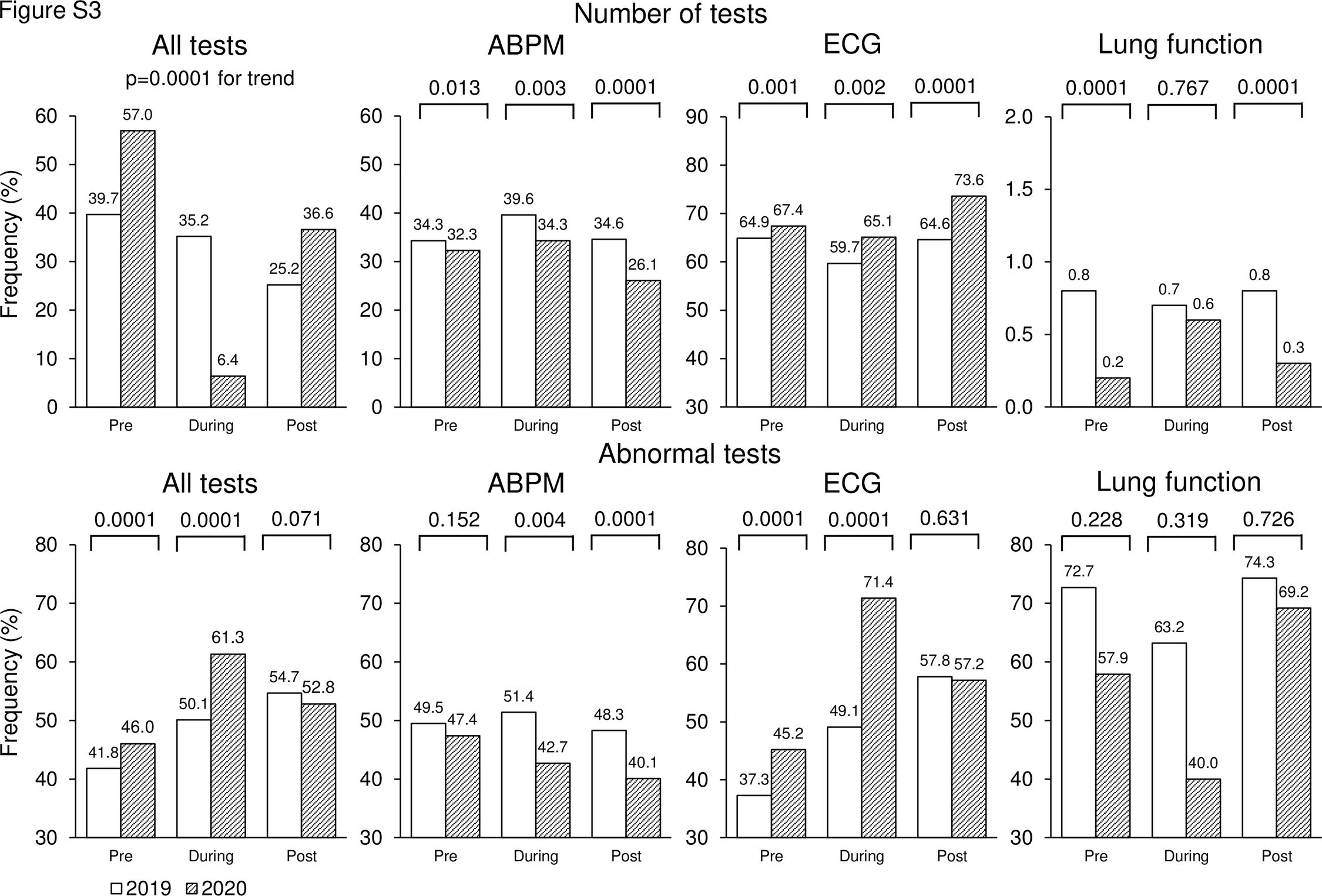

Supplement: S3 Fig — Pre-lockdown, lockdown and post-lockdown periods are identified. P-values of the differences are reported on top of each group of bars. ABPM: Ambulatory Blood Pressure Monitoring; ECG: Electrocardiogram. (TIF) [file pone.0258015.s003.tif]

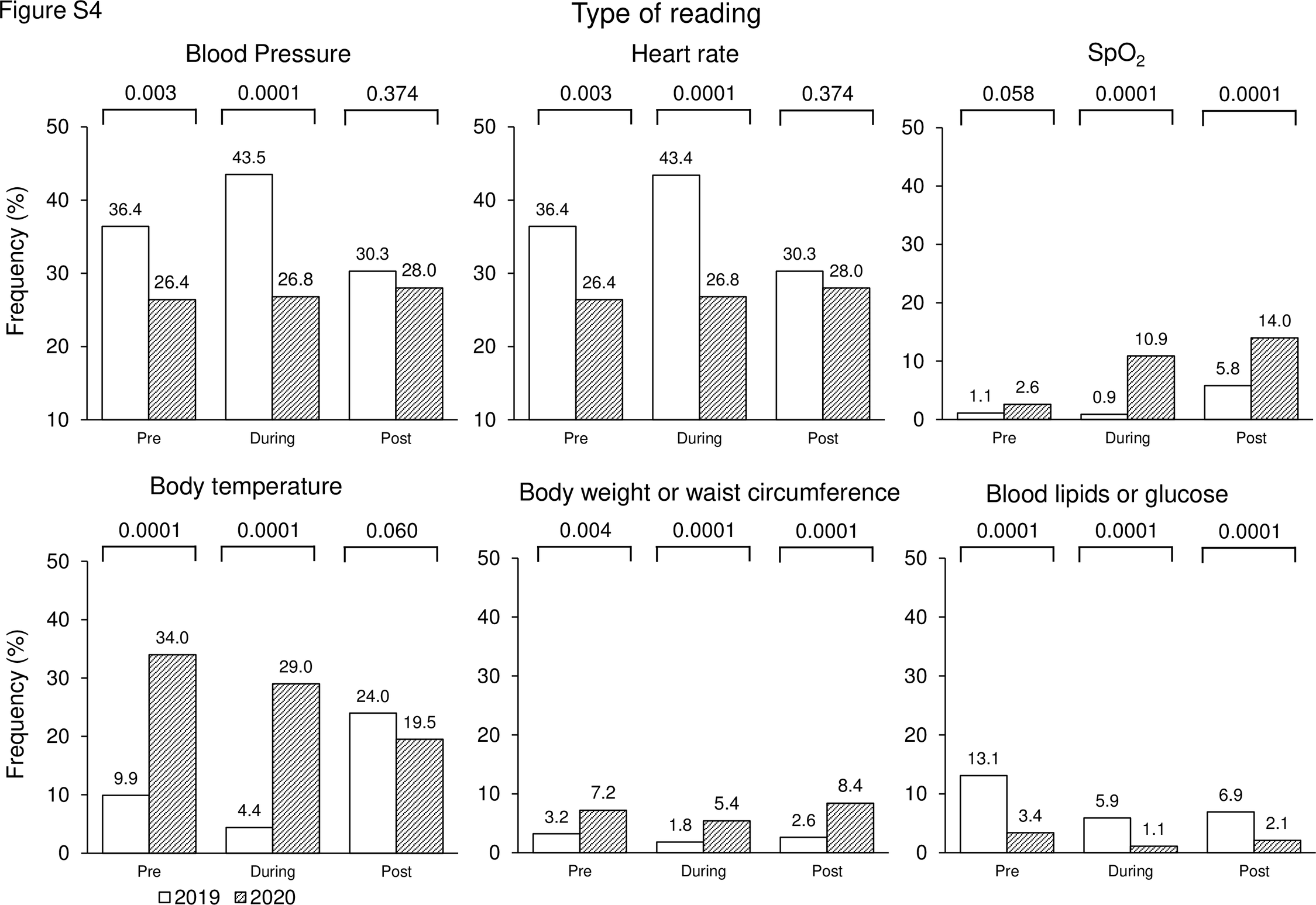

Supplement: S4 Fig — Pre-lockdown, lockdown and post-lockdown periods are identified. P-values of the differences are reported on top of each group of bars. (TIF) [file pone.0258015.s004.tif]

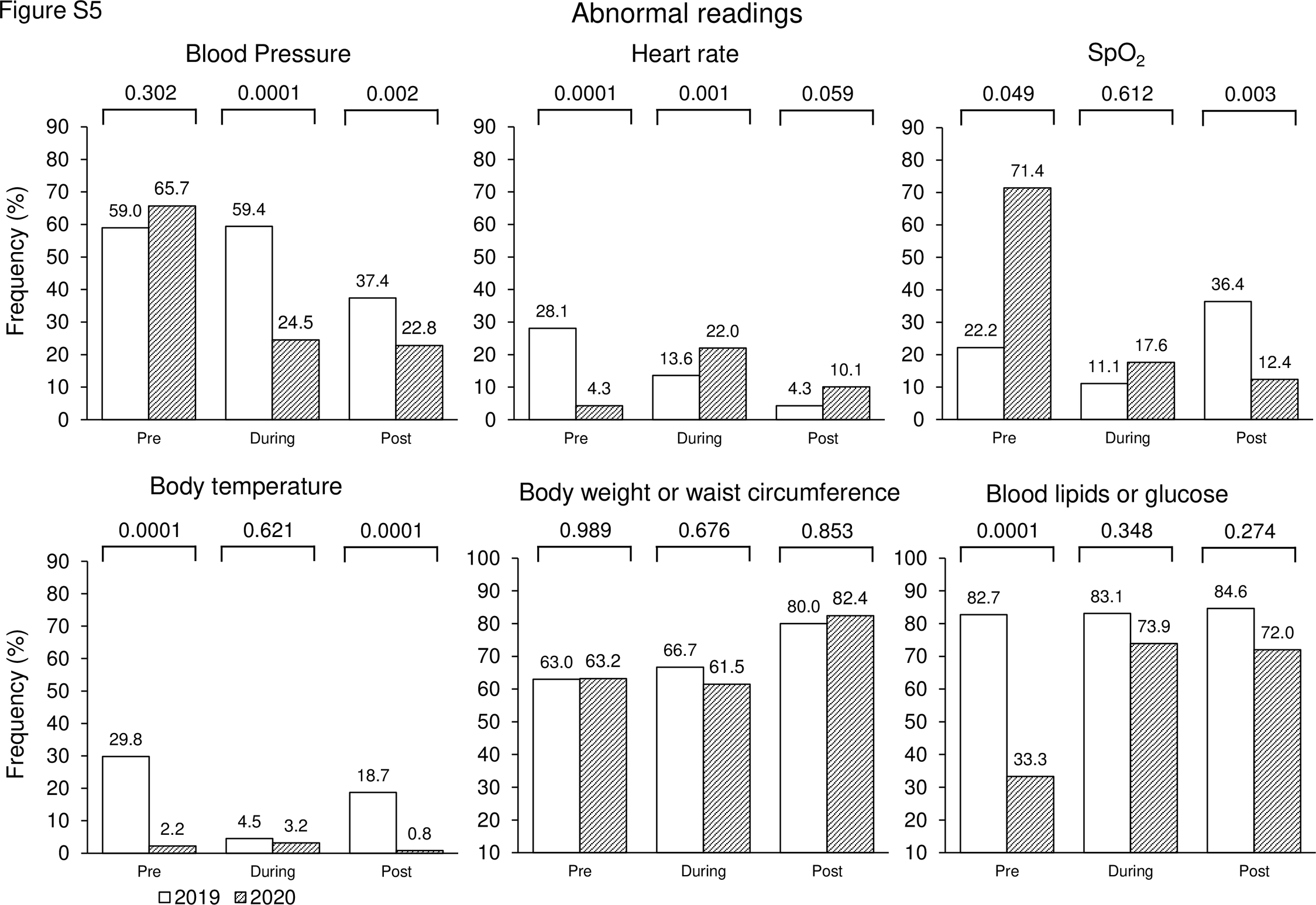

Supplement: S5 Fig — Pre-lockdown, lockdown and post-lockdown periods are identified. P-values of the differences are reported on top of each group of bars. (TIF) [file pone.0258015.s005.tif]
