# Supplementary material for: Telehealth at scale can improve chronic disease management in the community during a pandemic: An experience at the time of COVID-19
Source: PLoS One. 2021 Sep 29;16(9):e0258015. doi: 10.1371/journal.pone.0258015 (PMC8480747; doi:10.1371/journal.pone.0258015)
Supplement: S1 Table — Data are shown according to the setting (pharmacies or general practitioners’ offices vs. home) and period (before, during, and after the lockdown). (DOCX) [file pone.0258015.s006.docx]

**S1 Table.** General characteristics of subjects and tests performed in the community before (2019) and during (2020) the COVID-19 pandemic. Data are shown according to the setting (pharmacies or general practitioners’ offices vs. home) and period (before, during, and after the lockdown).

| **Pharmacies and general practitioners’ offices** | **Pre-lockdown** | | | **Lockdown** | | | **Post- lockdown** | | | **Overall p-value** |
| --- | --- | --- | --- | --- | --- | --- | --- | --- | --- | --- |
|  | **2019** | **2020** | **p-value** | **2019** | **2020** | **p-value** | **2019** | **2020** | **p-value** |  |
| Operators | 589 | 637 | - | 588 | 279 | - | 570 | 528 | - | 0.0001 |
| Total subjects (n) | 6511 | 7758 | - | 5773 | 874 | - | 4135 | 4981 | - | 0.0001 |
| Age (years, mean±SD) | 47.6 ± 23.0 | 48.2 ± 23.1 | 0.0001 | 51.3 ±22.1 | 58.2 ± 18.9 | 0.011 | 54.5 ± 22.1 | 55.3 ± 22.5 | 0.0001 | 0.0001 |
| Sex |  |  |  |  |  |  |  |  |  |  |
| *Male (n, %)* | *3024 (46.4)* | *3374 (43.5)* | *0.0001* | *2672 (46.3)* | *425 (48.6)* | *0.196* | *1967 (47.6)* | *2264 (45.5)* | *0.044* | 0.0001 |
| *Female (n, %)* | *3487 (53.6)* | *4384 (56.5)* |  | *3101 (53.7)* | *449 (51.4)* |  | *2168 (52.4)* | *2717 (54.5)* |  |  |
| Antihypertensive treatment (n, %) | 883 (13.6) | 1027 (13.2) | 0.572 | 992 (17.2) | 137 (15.7) | 0.268 | 583 (14.1) | 579 (11.6) | 0.0001 | 0.0001 |
| Cardiovascular disease (n, %) | 954 (14.7) | 1237 (15.9) | 0.033 | 965 (16.7) | 209 (23.9) | 0.0001 | 821 (19.9) | 1072 (21.5) | 0.051 | 0.0001 |
| Cardiovascular risk factors (n, %) | 1651 (25.4) | 2029 (26.2) | 0.279 | 1735 (30.1) | 305 (34.9) | 0.004 | 1254 (30.3) | 1487 (29.9) | 0.624 | 0.0001 |
| Concomitant diseases, symptoms or treatments (n, %) | 2282 (44.3) | 3498 (45.1) | 0.323 | 3020 (52.3) | 571 (65.3) | 0.0001 | 2304 (55.7) | 2821 (56.6) | 0.380 | 0.0001 |
| Area of the country |  |  |  |  |  |  |  |  |  |  |
| North (n, %) | 2689 (41.4) | 2957 (38.1) | 0.0001 | 2348 (40.8) | 313 (35.8) | 0.0001 | 1632 (39.5) | 1894 (38.0) | 0.005 | 0.0001 |
| Center (n, %) | 1018 (15.7) | 1344 (17.3) |  | 874 (15.1) | 99 (11.3) |  | 681 (16.5) | 735 (14.8) |  |  |
| South (n, %) | 2790 (42.9) | 3457 (44.6) |  | 2537 (44.1) | 462 (52.9) |  | 1817 (44.0) | 2352 (47.2) |  |  |
| Abnormal test results (n, %) | 2723 (41.8) | 3566 (46.0) | 0.0001 | 2895 (50.1) | 536 (61.3) | 0.0001 | 2260 (54.7) | 2628 (52.8) | 0.071 | 0.0001 |

Cont.Cont.

| **Home users** | **Pre-lockdown** | | | **Lockdown** | | | **Post- lockdown** | | | **Overall p-value** |
| --- | --- | --- | --- | --- | --- | --- | --- | --- | --- | --- |
|  | **2019** | **2020** | **p-value** | **2019** | **2020** | **p-value** | **2019** | **2020** | **p-value** |  |
| Total subjects (n) | 38 | 21 | - | 35 | 170 | - | 27 | 61 | - | 0.0001 |
| Transmitted readings (n) | 1186 | 350 | - | 1458 | 2838 | - | 503 | 1602 | - | 0.0001 |
| Age (years, mean±SD) | 52.3 ± 14.4 | 54.5 ± 14.9 | 0.585 | 52.4 ± 16.6 | 49.1 ± 14.0 | 0.227 | 46.9 ± 14.9 | 52.6 ± 17.4 | 0.147 | 0.127 |
| Sex |  |  |  |  |  |  |  |  |  |  |
| *Male (n, %)* | *27 (71.1)* | *15 (71.4)* | *0.976* | *27 (77.1)* | *116 (68.2)* | *0.296* | *18 (66.7)* | *46 (75.4)* | *0.396* | *0.833* |
| *Female (n, %)* | *11 (28.9)* | *6 (28.6)* |  | *8 (22.9)* | *54 (31.8)* |  | *9 (33.3)* | *15 (24.6)* |  |  |
| Concomitant diseases or treatments (n, %) | 8 (21.1) | 5 (23.8) | 0.807 | - | 31 (18.2) | 0.006 | - | 10 (16.4) | 0.025 | 0.0001 |
| Abnormal values (n, %) | 489 (41.2) | 94 (26.9) | 0.0001 | 522 (35.8) | 510 (18.0) | 0.0001 | 111 (22.1) | 265 (16.5) | 0.005 | 0.0001 |
