# Supplementary material for: Telehealth at scale can improve chronic disease management in the community during a pandemic: An experience at the time of COVID-19
Source: PLoS One. 2021 Sep 29;16(9):e0258015. doi: 10.1371/journal.pone.0258015 (PMC8480747; doi:10.1371/journal.pone.0258015)
Supplement: S2 Table — (DOCX) [file pone.0258015.s007.docx]

**S2 Table.** General characteristics of subjects and tests performed in the community before (2019) and during (2020) the COVID-19 pandemic.

|  | **Pre-lockdown** | | | **Lockdown** | | | **Post-lockdown** | | | **Overall p-value** |
| --- | --- | --- | --- | --- | --- | --- | --- | --- | --- | --- |
|  | **2019** | **2020** | **p-value** | **2019** | **2020** | **p-value** | **2019** | **2020** | **p-value** |  |
| **ECG** | 4223 | 5231 |  | 3447 | 569 |  | 2671 | 3666 |  |  |
| Age (years, mean±SD) | 41.8 ± 24.6 | 43.0 ± 24.7 | 0.013 | 46.4 ± 24.7 | 57.8 ± 20.6 | 0.0001 | 51.7 ± 24.6 | 53.2 ± 24.0 | 0.012 | 0.0001 |
| Sex |  |  |  |  |  |  |  |  |  |  |
| Male (n, %) | 1903 (45.1) | 2254 (43.1) | 0.055 | 1608 (46.6) | 271 (47.6) | 0.665 | 1292 (48.4) | 1701 (46.4) | 0.120 | 0.0001 |
| Female (n, %) | 2320 (54.9) | 2977 (56.9) |  | 1839 (53.4) | 298 (52.4) |  | 1379 (51.6) | 1965 (53.6) |  |  |
| Concomitant diseases, symptoms or treatments (n, %) | 1655 (39.4) | 2080 (39.8) | 0.740 | 1689 (49.0) | 398 (69.9) | 0.0001 | 1508 (56.5) | 2024 (55.2) | 0.323 | 0.0001 |
| Diagnosis |  |  |  |  |  |  |  |  |  |  |
| Normal | 2594 (61.4) | 2713 (51.9) | 0.0001 | 1701 (49.3) | 138 (24.3) | 0.0001 | 1099 (41.1) | 1423 (38.8) | 0.061 | 0.0001 |
| Arrhythmias | 115 (2.7) | 193 (3.7) | 0.009 | 119 (3.5) | 31 (5.4) | 0.020 | 116 (4.3) | 152 (4.1) | 0.701 | 0.0001 |
| Conduction abnormalities | 256 (6.1) | 638 (12.2) | 0.0001 | 354 (10.3) | 51 (9.0) | 0.338 | 243 (9.1) | 386 (10.5) | 0.060 | 0.0001 |
| Hypertrophy | 9 (0.2) | 28 (0.5) | 0.013 | 13 (0.4) | 1 (0.2) | 0.450 | 9 (0.3) | 18 (0.5) | 0.353 | 0.128 |
| Ischemia | 1249 (29.6) | 1659 (31.7) | 0.025 | 1260 (36.6) | 348 (61.2) | 0.0001 | 1204 (45.1) | 1687 (46.0) | 0.458 | 0.0001 |
| **ABPM** | 2233 | 2508 |  | 2288 | 300 |  | 1429 | 1302 |  |  |
| Age (years, mean±SD) | 58.4 ± 14.5 | 58.9 ± 14.7 | 0.253 | 58.7 ± 14.6 | 59.1 ± 15.2 | 0.665 | 59.6 ± 15.3 | 61.8 ± 15.9 | 0.005 | 0.0001 |
| Sex |  |  |  |  |  |  |  |  |  |  |
| Male (n, %) | 1082 (48.5) | 1108 (44.2) | 0.003 | 1039 (45.4) | 150 (50.0) | 0.134 | 648 (45.3) | 556 (42.7) | 0.165 | 0.006 |
| Female (n, %) | 1151 (51.5) | 1400 (55.8) |  | 1249 (54.6) | 150 (50.0) |  | 781 (54.7) | 746 (57.3) |  |  |
| Antihypertensive treatment (n, %) | 883 (34.5) | 1027 (40.9) | 0.325 | 992 (43.4) | 137 (45.7) | 0.448 | 583 (40.8) | 579 (44.5) | 0.053 | 0.016 |
| Concomitant diseases, symptoms or treatments (n, %) | 1207 (54.1) | 1407 (56.1) | 0.157 | 1322 (57.8) | 171 (57.0) | 0.797 | 783 (54.8) | 792 (60.8) | 0.001 | 0.002 |
| Day+night time hypertension | 928 (41.6) | 914 (38.2) | 0.019 | 970 (42.4) | 89 (32.6) | 0.002 | 535 (37.4) | 381 (30.8) | 0.0001 | 0.0001 |
| Day-time hypertension with night-time normotension | 205 (9.2) | 221 (9.2) | 0.952 | 226 (9.9) | 15 (5.5) | 0.019 | 66 (4.6) | 39 (3.2) | 0.052 | 0.0001 |
| Night-time hypertension with day-time normotension | 352 (15.8) | 330 (13.8) | 0.058 | 352 (15.4) | 43 (15.8) | 0.874 | 331 (23.2) | 286 (23.1) | 0.978 | 0.0001 |
